# PD-L1 and VEGF dual blockade enhances anti-tumor effect on brain metastasis in hematogenous metastasis model

**DOI:** 10.1007/s10585-024-10309-y

**Published:** 2024-09-05

**Authors:** Chinami Masuda, Shinichi Onishi, Keigo Yorozu, Mitsue Kurasawa, Mamiko Morinaga, Daiko Wakita, Masamichi Sugimoto

**Affiliations:** Product Research Department, Chugai Pharmaceutical Co., Ltd., Chugai Life Science Park Yokohama, 216, Totsuka-Cho, Totsuka-Ku, Yokohama, Kanagawa 244-8602 Japan

**Keywords:** Anti-PD-L1 antibody, Anti-VEGF antibody, Hematogenous brain metastasis model, Immunotherapy

## Abstract

Immunotherapy improves survival outcomes in cancer patients, but there is still an unmet clinical need in the treatment of brain metastases. Here, we used a mouse model to investigate the antitumor effect of programmed death-ligand 1 (PD-L1) and vascular endothelial growth factor (VEGF) dual blockade on metastatic brain tumors and evaluated immune responses during treatment. After establishing hematogenous brain metastasis by transplanting murine bladder carcinoma MBT2 cells stably expressing secNLuc reporter via the internal carotid artery of C3H/HeNCrl mice, we observed the formation of metastases not only in the brain parenchyma but also in the ventricles. The observed pathological areas showed that metastases in the ventricle were histologically larger than that in the brain parenchyma. Regarding the total tumor burden in the whole brain as revealed by Nluc activities, the combination of anti-PD-L1 antibody and anti-VEGF antibody showed a stronger anti-tumor effect than each single agent. Anti-PD-L1 antibody alone enhanced CD8^+^ T cell priming in regional lymph nodes, increased the proportion of activated CD8^+^ T cells in whole brain, and increased the density of CD8^+^ cells in the brain parenchyma. Furthermore, anti-VEGF antibody alone decreased microvessel density (MVD) in ventricular metastases, and the combination treatment increased intratumoral CD8^+^ cell density in the brain parenchyma and ventricular metastases. These results suggest that PD-L1 blockade enhanced cancer immunity not only in brain metastases lesions but also in the regional lymph nodes of the metastases, and that the addition of VEGF blockade increased the antitumor effect by increasing the infiltration of activated CD8^+^ T cell and decreasing MVD.

**Supplementary Information:**

The online version contains supplementary material available at 10.1007/s10585-024-10309-y.

## Introduction

Brain metastases are a common complication of solid tumors and a leading cause of neurologic impairments and death in cancer patients [1]. The most frequent intracranial metastatic site is the brain parenchyma [2]. Surgical resection and radiation therapy are used for local control of brain metastases, but their therapeutic effects are limited. Therefore, more effective pharmacological intervention treatments are eagerly awaited.

Programmed death-ligand 1 (PD-L1) is expressed on tumor cells and immune cells and is a primary ligand for an immune checkpoint molecule programmed death-1 (PD-1). Anti-PD-L1 antibody inhibits the interaction between PD-L1 and PD-1 on T cells in lymph nodes and tumors and releases T cell suppression, and thus promotes the proliferation and activation leading to anti-tumor effects [3, 4].

Vascular endothelial growth factor (VEGF) is one of the key angiogenic factors in tumors and participates in the initial stage of tumor development, progression, and metastasis [5]. Anti-VEGF antibody targets VEGF, causing the shrinkage of existing tumor blood vessels and the inhibition of angiogenesis demonstrating anti-tumor activity [6, 7]. In addition to quantitative changes in blood vessels, various positive effects on tumor immunity have also been reported, such as the maturation of dendritic cells (DC), the promotion of T cell infiltration into tumors, and the inhibition of regulatory T (Treg) cells and myeloid derived suppressor cells (MDSC) by anti-VEGF antibodies [8–14]. These effects provide the rationale for its combination with immune checkpoint inhibitors (ICIs).

Indeed, treatment regimens including combinations of anti-PD-1/PD-L1 antibodies and VEGF antibodies have been approved for use in clinical settings for non-squamous non-small cell lung cancer (NSCLC) and hepatocellular carcinoma [15, 16]. Moreover, many clinical trials are currently underway, making it one of the most promising combinations in cancer immunotherapy [17, 18].

The effect of ICIs on metastatic tumors, including brain metastasis and their mechanisms, remains poorly understood. Firstly, the presence of the Blood–Brain Barrier (BBB), which restricts the transport of substances to the brain, is thought to potentially limit the penetration of systemic therapeutic drugs. However, recent reports have shown that the BBB changes to the Blood–Tumor Barrier (BTB) during the development of primary or metastatic brain tumors [19, 20]. Secondly, the semi-privileged immune environment created by the BTB may limit the infiltration and maintenance of immune cells in the microenvironment of brain metastasis. Recent single-cell based integrative analysis of transcriptomics and genetics have characterized the tumor immune microenvironment of brain metastases, and there are increasing reports of immune cell infiltration in brain metastases [21–26]. On the other hand, there is limited evidence showing that immune cells infiltrating brain metastases are directly involved in the shrinkage of brain metastases following treatment with ICIs. It is important to confirm the above mechanism using an appropriate model, and there are variety of reported brain metastasis models. Of these, the hematogenous model, which is improved by the intracarotid inoculation of tumor cells, is known to recapitulate the later steps in tumor brain metastasis, e.g., adhesion to the brain vasulature, extravasation, and outgrowth in a brain-specific environment [27]. In our prior research, we developed a stable model that enables to assess drug efficacy by transplanting human tumor cell lines into the internal carotid artery of immunodeficient mice, which formed cerebral metastases. We showed that systemically administered anti-VEGF antibodies penetrate these brain metastatic lesions and exert anti-tumor activity, accompanied by a decrease in tumor microvessel density (MVD) [28]. Furthermore, we modified this model using an adoptive immunization approach, transferring immune cells from human cell line-immunized mice to immunodeficient mice with established cerebral metastases. We demonstrated that the transferred CD8^+^ T cells infiltrate the brain metastases, and that the systemic administration of anti-PD-L1 antibodies promotes the activation of CD8^+^ T cells, significantly enhancing the anti-tumor effect against brain metastases [29].

In the present study, we used immune-competent mice with the aim of deeply and precisely analyzing the anti-tumor immunity against brain metastases. We formed brain metastases by transplanting syngeneic mouse tumor cells via the internal carotid artery, elucidated the antitumor efficacy of the combined use of anti-PD-L1 and anti-VEGF antibodies on established brain metastases in immune-competent mice, and performed an examination of the immune cells within the brain metastases and the associated lymph nodes, as well as an assessment of the tumor blood vessel density.

## Materials and methods

### Reagents

Monoclonal murine anti-PD-L1 and anti-VEGF antibodies were provided by Genentech (South San Francisco, CA, USA). The monoclonal murine anti-PD-L1 antibody (mAb; clone 6E11) and anti-VEGF antibody (mAb; clone B20-4.1.1) have the capability to bind both human and mouse PD-L1 and VEGF, respectively. Mouse Immunoglobulin G (IgG) was purchased from SouthernBiotech (Birmingham, AL, USA). All reagents were diluted in saline to achieve the desired concentrations for the respective assays.

### Cell lines and culture conditions

MBT2 murine bladder carcinoma cells (JCRB No. IFO50041, established by Soloway, M. S.) were obtained from the Japanese Collection of Research Bioresources (JCRB; Osaka, Japan) [30]. It was maintained in E-MEM (Sigma-Aldrich, Tokyo, Japan) supplemented with 10% (v/v) fetal bovine serum (Sigma-Aldrich).

The MBT2 secNluc cell line stably expressed the luciferase reporter gene secNluc. The CAG-secNluc sequence from the pEBMulti-secNluc vector, which was prepared as described previously [28, 29], was amplified using the PCR method. This PCR product then was integrated into the pLenti6/V5-D-TOPO vector (Thermo Fisher Scientific Inc, Waltham, MA, USA), from which the CMV has been removed, to construct the pLenti6-CAG-secNluc vector. Subsequently, this constructed vector was introduced into 293FT cells using the ViraPower system (Thermo Fisher Scientific Inc) to generate lentivirus. This virus was then used to infect MBT2 cells, establishing those that stably express secNluc. Stable cells were selected and maintained in the culture medium containing Blasticidin (Thermo Fisher Scientific Inc) at 37 °C under 5% CO_2_.

### Laboratory animals

Female C3H/HeNCrl mice (7–8-weeks old; The Jackson Laboratory Japan, Inc., Kanagawa, Japan) were used. All animal experiments were reviewed and approved by the Institutional Animal Care and Use Committee at Chugai Pharmaceutical Co., Ltd., and conformed to the Guide for the Care and Use of Laboratory Animals published by the Institute of Laboratory Animal Resources.

### Preparation of a brain metastasis model and in vivo treatment

The brain metastasis model was created by applying the technique used to establish a hematogenous metastasis model through transplantation via the internal carotid artery in immune-competent mice. In brief, C3H/HeNCrl mice were anesthetized by isoflurane. In addition to the external carotid artery, the occipital artery and pterygopalatine artery were ligated and the catheter was inserted from the common carotid artery towards the internal carotid artery. Then, MBT2 secNluc cells (1 × 10^5^ cells/head) were injected slowly into the internal carotid artery through the inserted catheter, and finally the cut made in the skin was stitched up. One week after tumor inoculation, blood was collected from the jugular vein and Nluc activity in plasma was measured and used to randomize mice into control and test groups (day (1). An intraperitoneal administration of mouse IgG and anti-PD-L1 antibody was carried out twice a week at a dose of 10 mg/kg. Similarly, mouse IgG and anti-VEGF antibody were administered intraperitoneally at a dose of 10 mg/kg once a week. Mice that showed significant weight loss and weakness accompanied by loss of locomotor activity were euthanized.

### Analysis of Nluc activity

Blood was collected from the jugular vein of the mouse, and plasma was collected after centrifugation and used for analyses. Brains were removed from mice after euthanasia, frozen immediately in liquid nitrogen, and stored at − 80 °C until use. The brains were homogenized in cell lysis buffer (Cell Signaling Technology, Danvers, MA, USA) and the supernatant in each homogenate was collected after centrifugation and used for analyses. Luminescence was measured using Nano-Glo® Luciferase Assay System (Promega Corporation, Madison, WI, USA) according to the manufacturer’s instructions. A Varioskan Plate reader (Thermo Fisher Scientific Inc) was used to measure the luminescence.

### Flow cytometry (FCM) analysis

Brains were excised after euthanasia and single-cell suspensions were obtained by mincing brain and digestion with a gentleMACS Dissociator and mouse tumor dissociation kit (Miltenyi Biotec, Bergisch Gladbach, Germany). Debris Removal Solution (Miltenyi Biotec) was then used to remove debris according to the manufacturer’s protocol.

Deep and superficial cervical lymph nodes (CLNs) were removed under a surgical microscope after euthanasia and minced CLNs were digested with Collagenase D (Sigma-Aldrich) while shaken with rotation. Single-cell suspensions were prepared by passing through a cell strainer.

Single-cell suspensions were incubated with anti-Fcγ receptor antibody (Clone 2.4G2, BD Biosciences, San Jose, CA, USA) and the fixable viability dye FVD780 (Thermo Fisher Scientific Inc), then stained with the following monoclonal antibodies: peridinin-chlorophyll-protein complex (PerCP)-Cy5.5–CD45 (30-F11), brilliant ultra violet (BUV)737-CD3 (17A2), BUV395-CD4 (GK1.5), brilliant violet (BV)510-CD11b (M1/70), phycoerythrin (PE)-CF594–F4/80 (T45-2342), BUV395-major histocompatibility complex class II (MHC-II, 2G9), Alexa700-granzyme B (GB11), BV786-Ki67 (B56), PE-CD274 (PD-L1, MIH5), from BD Biosciences, BV510-CD45 (30-F11), PE-Cy7-CD335(NKp46, 29A1.4), Alexa Fluor 700-Ly-6G (Gr-1, RB6-8C5), BV711-CD11c (N418), BV650-CD69 (H1.2F3), BV421-CD279 (PD-1, 29F.1A12), PE-Cy7-CD80 (16-10A1), BV605-CD86 (GL-1), Alexa647-CD197 (CCR7, 4B12), from BioLegend (San Diego, CA, USA), fluorescein isothiocyanate (FITC)-CD8 (KT15) from Medical & Biological Laboratories (Tokyo, Japan), PerCP-Cy5.5-Foxp3 (FJK-16s) from Thermo Fisher Scientific.

The appropriate conjugated isotype-matched IgG was used as the control when necessary. Intracellular staining was performed using a Foxp3/Transcription Factor Staining Buffer Set (Thermo Fisher Scientific Inc). Stained cells were analyzed using a LSRFortessa X-20 cell analyzer (BD Biosciences) and data analyzed with FlowJo 10 software (BD, Franklin Lakes, NJ, USA). The gating strategies are shown in Supplementary Fig. S1, S2, S3 andS4 .

### Immunohistochemistry (IHC) analysis

Brains were excised after euthanasia and fixed in 10% Neutral Buffered Formalin. After dividing the brains into six parts, paraffin-embedded sections were sliced to 4 μm thickness, stained with the VENTANA automated slide stainers (Ventana Medical Systems Inc, Oro Valley, AZ, USA), and visualized using light microscopy. The localization of CD8^+^ cells and GzmB^+^ cells in the brain was evaluated by immunohistochemical staining of CD8 using CD8α (D4W2Z) XP® Rabbit mAb and GzmB using GzmB (E5V2L) Rabbit mAb, both from Cell Signaling Technology. The detection of CD31^+^ cells in the brain was assessed by IHC staining of CD31 using CD31 Rabbit mAb from Arigo Biolaboratories (Hsinchu, Taiwan ROC). Secondary antibodies were used OptiView DAB IHC Detection Kit (Roche Diagnostics, Rotkreuz, Switzerland).

Tumors were detected using a deep learning tissue classification model　with imaging analysis software HALO AI v3.4.2986.209 (Indica labs, Albuquerque, NM, USA). We conducted quantitative analysis of CD8^+^ and GzmB^+^ cell density within the tumor in brain ventricular metastases and within the tumor and the peritumoral area within 500 μm of the tumor border in brain parenchymal metastases. We measured the microvessel density (MVD) based on CD31^+^ area ratio in the ventricular metastatic lesions using an image analysis algorithm with HALO AI v3.4.2986.209.

### Statistical analysis

For multiple comparisons, data were analyzed with the Wilcoxon rank sum test, and then the P-values were corrected using the Holm-Bonferroni method. Corrected P-values < 0.05 were considered to indicate a statistically significant difference. All statistical analyses were conducted using JMP® Version 15 software (SAS Institute Inc., Cary, NC, USA).

## Results

### Pathological analysis of brain metastases

**Fig. 1:**
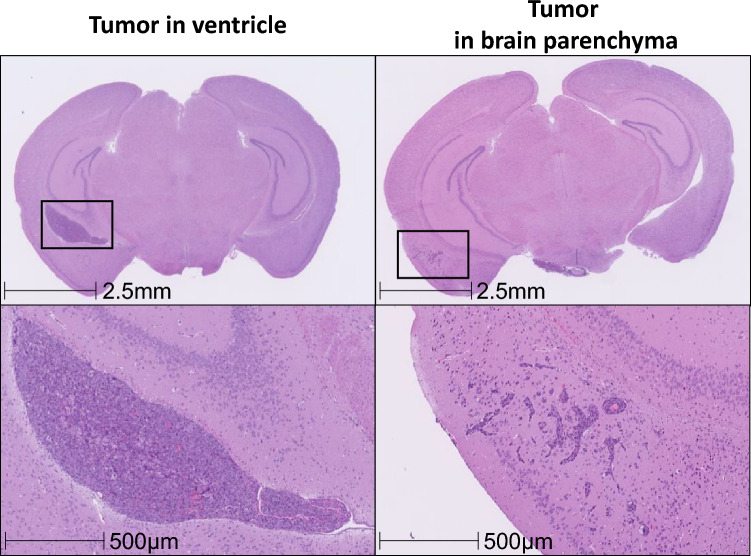
Profile of the hematogenous brain metastasis model. MBT2 secNluc cell transplantation into the internal carotid artery of C3H/HeNCrl mice. Brain samples were collected on day 14 after MBT2 secNluc inoculation. Representative micrographs of HE staining. Scale bar is 2.5 mm and 500 μm at higher magnification. Evaluation of metastatic foci in the ventricles and brain parenchyma was determined by a pathologist

After the establishment of hematogenous brain metastasis by transplanting the MBT2 secNluc cells via the internal carotid artery, the brain was divided into 6 sections using a brain slicer, and the brain metastatic foci on the 6 sections were histopathologically analyzed. In this model, metastatic foci were observed not only in the brain parenchyma but also in the ventricles. Ventricular metastases proliferate compressively and had distinct borders while parenchymal metastases proliferate invasively. The area of the ventricular metastatic foci was histologically larger than those in the brain parenchyma (Fig. 1).

### The effects of anti-PD-L1 antibody in combination with anti-VEGF antibody on the brain metastasis

The antitumor effect of antibodies was evaluated as total tumor burden in the whole brain using Nluc activity, reflecting the sum of all parts of the entire intracranial metastases, including the brain parenchyma and ventricles.

**Fig. 2:**
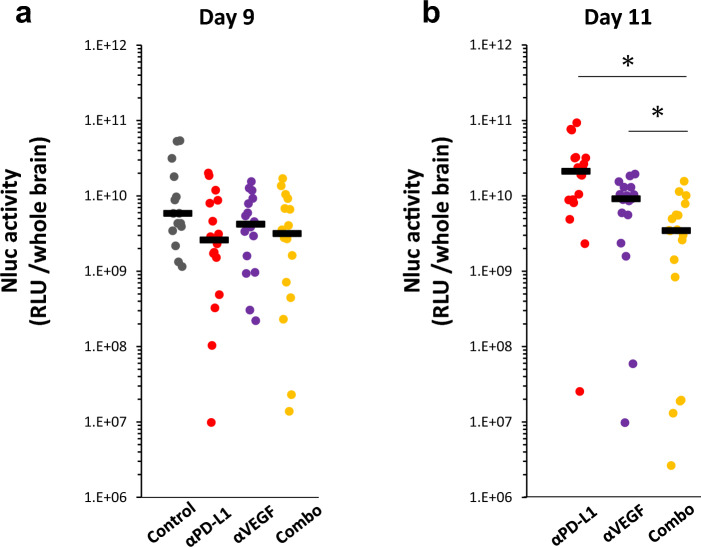
Antitumor effects of anti-PD-L1 antibody in combination with anti-VEGF antibody on established brain metastasis. Anti-PD-L1 antibody (αPD-L1) or mouse IgG (Control) was administered intraperitoneally into a brain metastasis model mice twice a week at a dose of 10 mg/kg. The anti-VEGF antibody (αVEGF) or mouse IgG (Control) was administered intraperitoneally into brain metastasis model mice weekly at a dose of 10 mg/kg. Brain samples were collected on day 9 (4 group study) or day 11 (3 group study), with the drug efficacy study starting on day 1. Two or three independent experiments were pooled and analyzed. Antitumor activity on brain metastasis was evaluated by measuring Nluc activity (relative light unit /whole brain) in the supernatant of brain homogenates. **a** Antitumor activity on day 9 (n = 15 or 16/group). **b** Antitumor activity on day 11 (n = 17 or 18/group). Dots indicate individuals and bars represent median. Statistical differences are shown as *, p < 0.05 (assessed by Wilcoxon’s rank sum test with Holm-Bonferroni correction)

The comparative analysis of the antitumor effect on brain metastasis was conducted by evaluating the Nluc activity in the brains of mice treated with control IgG (Control), anti-PD-L1 antibody (anti-PD-L1), anti-VEGF antibody (anti-VEGF), and a combination of anti-PD-L1 and anti-VEGF (Combo). On day 9, a trend towards decreased brain Nluc activity was observed in the drug administration group, compared to control but was not statistically significant at this time point (Fig. 2a). Since some IgG-treated mice reached the euthanasia threshold after day 9, a comparison of the drug groups was conducted excluding the control group. Based on whole brain Nluc activity on day 11, the combination of anti-PD-L1 and anti-VEGF showed stronger anti-tumor effect than each single agent (Fig. 2b).

### ***Treatment with anti-PD-L1 antibody enhanced priming and activation of CD8***^+^***T cells, CD4***^+^***T cells, and DC in CLNs***

Next, we investigated the underlying mechanism by examining the effects of anti-PD-L1 and anti-VEGF treatment on the activation of immune cells in the whole brain and in the CLNs, which have been reported as draining lymph nodes for brain tumors, including brain metastatic lesions, and as draining lymph nodes for cerebrospinal fluid filling the lumen of the ventricles [31–34].

**Fig. 3:**
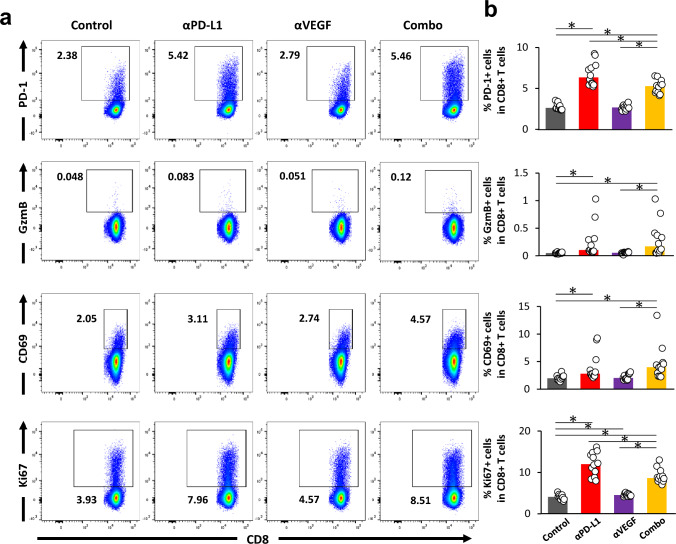

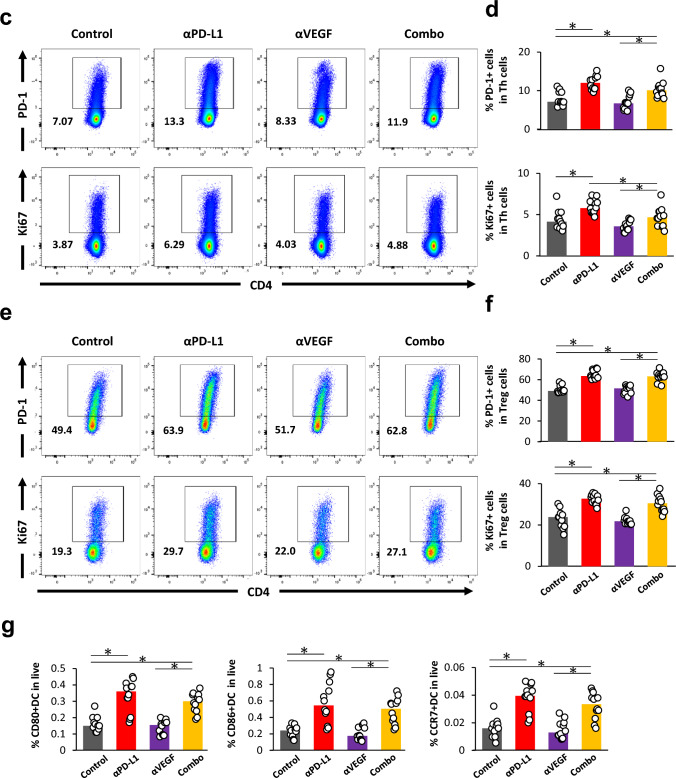
Treatment with anti-PD-L1 antibody enhanced priming and activation in CLNs. Anti-PD-L1 antibody (αPD-L1) or mouse IgG (Control) was administered intraperitoneally into a brain metastasis model mice twice a week at a dose of 10 mg/kg. The anti-VEGF antibody (αVEGF) or mouse IgG (Control) was administered intraperitoneally into brain metastasis model mice weekly at a dose of 10 mg/kg. Deep and superficial cervical lymph nodes (CLNs) were removed from the mice on day 8, with the drug efficacy study starting on day 1 (n = 11 or 12/group). Three independent experiments were pooled and analyzed. Proportion of activated CD8^+^ T cells, conventional helper T cells, regulatory T cells, and Dendritic cells in CLNs were analyzed using flow cytometry. **a** Representative flow cytometric profiles of CD8^+^ T cells expressing PD-1, Granzyme B, CD69, and Ki-67. **b** Proportion of PD-1^+^ cells, Granzyme B^+^ cells, CD69^+^ cells, and Ki-67^+^ cells in CD8^+^ T cell. **c** Representative flow cytometric profiles of conventional helper T cells expressing PD-1 and Ki-67. **d** Proportion of PD-1^+^ cells and Ki-67^+^ cells in conventional helper T cells. **e** Representative flow cytometric profiles of regulatory T cells expressing PD-1 and Ki-67. **f** Proportion of PD-1^+^ cells and Ki-67^+^ cells in regulatory T cells. **g** Proportion of CD80^+^/CD86^+^/CCR7.^+^ DC in live cells. Dots indicate individuals and bars represent median. Statistical differences are shown as *, p < 0.05 (assessed by Wilcoxon’s rank sum test with Holm-Bonferroni correction)

First, the number and activation status of immune cells in CLNs were analyzed using FCM (Fig. 3a). We found that higher percentages of CD8^+^ T cell expressing activation markers such as PD-1, GzmB, CD69, and activation/proliferation marker Ki-67 were observed with the anti-PD-L1 treatment compared to control. There was no significant difference between anti-VEGF alone and control except for Ki-67. Although there was no significant difference in the proportion of GzmB- and CD69-expressing cells in the combination group compared with anti-PD-L1 alone, a decrease in the proportion of PD-1- and Ki-67- expressing cells was observed (Fig. 3b).

In addition to the role of CD8^+^ T cells, mounting evidence points to the critical role of CD4^+^ T cells in antitumor immune responses [35]. Accordingly, we investigated the effects of these treatments on CD4^+^ T cells (Fig. 3c, e). Higher percentages of CD4^+^ FOXP3^−^ conventional helper T cells (Thconv) expressing PD-1 and Ki-67 were observed with the anti-PD-L1 treatment compared to control (Fig. 3d). Interestingly, higher percentages of CD4^+^ FOXP3^+^ Treg cells expressing PD-1 and Ki-67 with anti-PD-L1 were also observed (Fig. 3f). There was no change with anti-VEGF alone (Fig. 3d, f).

Dendritic cells (DCs) are functionally specialized myeloid-derived antigen-presenting cells that play an important role in priming tumor antigen-specific T cells in tumor-draining lymph nodes [36], and some reports suggesting that VEGF could limit the functional maturation of DCs. Clinical data from patients treated with bevacizumab show an increase in the number of mature DCs (maturation markers, CD80, CD83, and CD86) in peripheral blood in response to treatment [37]. Therefore, we investigated the functional maturation of DC by administering anti-VEGF. We confirmed the expression of PD-L1 on DCs (Gr-1^−^ F4/80^−^ CD11c^+^ MHC class II^+^ cells) in CLNs in control in this model using FCM analysis (Supplementary Fig. S1). Although the percentage of CD80^+^ and CD86^+^ DC in live cells was not changed by administering anti-VEGF, their number was increased with anti-PD-L1 compared to control. There was no change with anti-VEGF alone, and the above changes seen with anti-PD-L1 were also observed even in the presence of the anti-VEGF combination, but no difference was observed compared with anti-PD-L1 alone (Fig. 3g).

Taking together, the anti-PD-L1 treatment increased the activation of CD8^+^ T cells in CLNs, suggesting the augmentation of priming in the draining lymph nodes of the brain-metastasis lesions. The percentages of activated Thconv and Treg cells were increased by anti-PD-L1, and the percentage of activated DCs in live cells was increased by anti-PD-L1. Anti-VEGF did not affect the activation status of these cells in CLNs.

### ***Treatment with anti-PD-L1 antibody enhanced activation of CD8***^+^***T cells in metastatic brain***

**Fig. 4:**
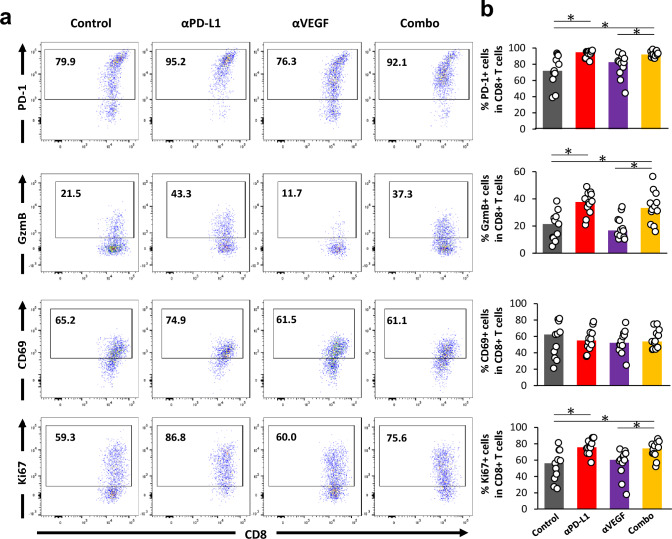

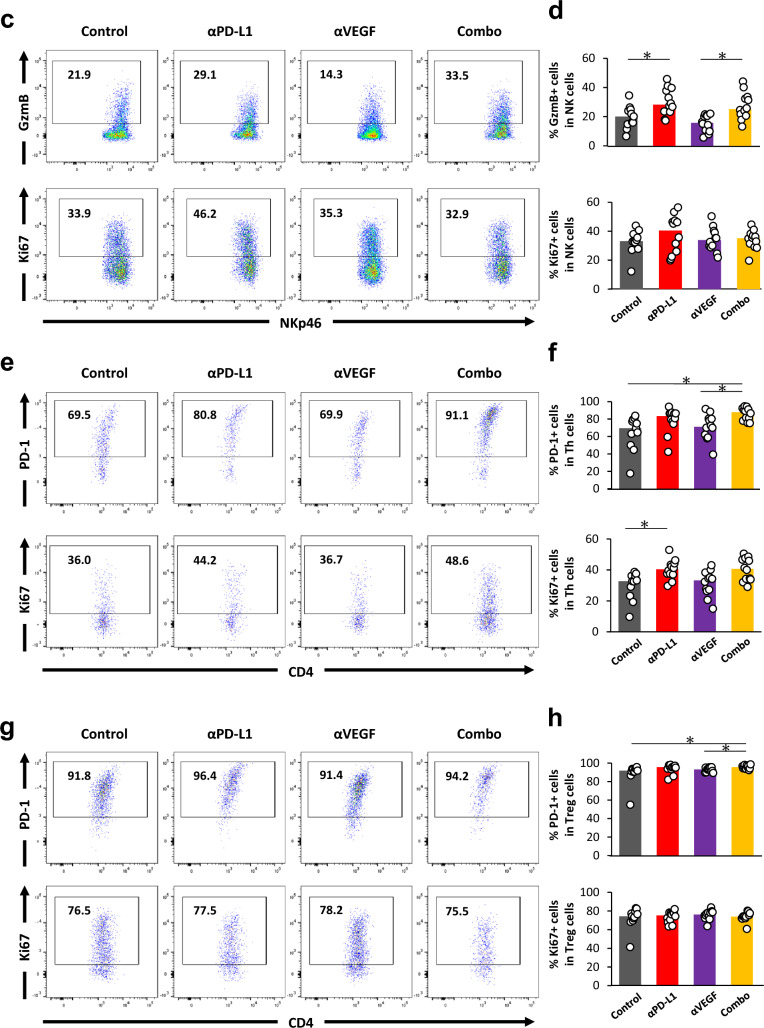
Treatment with anti-PD-L1 antibody enhanced activation of CD8^+^ T cells in the metastatic brain. Anti-PD-L1 antibody (αPD-L1) or mouse IgG (Control) was administered intraperitoneally into a brain metastasis model mice twice a week at a dose of 10 mg/kg. The anti-VEGF antibody (αVEGF) or mouse IgG (Control) was administered intraperitoneally into brain metastasis model mice weekly at a dose of 10 mg/kg. Brains were removed from the mice on day 8, with the drug efficacy study starting on day 1 (n = 11 or 12/group). Three independent experiments were pooled and analyzed. Proportion of activated CD8^+^ T cells, natural killer cells, conventional helper T cells, and regulatory T cells in whole brain were analyzed using flow cytometry. **a** Representative flow cytometric profiles of CD8^+^ T cells expressing PD-1, Granzyme B, CD69, and Ki-67. **b** Proportion of PD-1^+^ cells, Granzyme B^+^ cells, CD69^+^ cells, and Ki-67^+^ cells in CD8^+^ T cell. **c** Representative flow cytometric profiles of natural killer cells expressing Granzyme B and Ki-67. **d** Proportion of Granzyme B^+^ cells and Ki-67^+^ cells in natural killer cells. **e** Representative flow cytometric profiles of conventional helper T cells expressing PD-1 and Ki-67. **f** Proportion of PD-1^+^ cells and Ki-67^+^ cells in conventional helper T cells. **g** Representative flow cytometric profiles of regulatory T cells expressing PD-1 and Ki-67. **h** Proportion of PD-1^+^ cells and Ki-67^+^ cells in regulatory T cells. Dots indicate individuals and bars represent median. Statistical differences are shown as *, p < 0.05 (assessed by Wilcoxon’s rank sum test with Holm-Bonferroni correction)

Next, we analyzed the activation of each immune cell type in the whole brain using FCM (Fig. 4a). Compared to control, the administration of anti-PD-L1 increased percentages of CD8^+^ T cells expressing PD-1, GzmB, and Ki-67. There was no significant difference with anti-VEGF alone, and the above changes seen with anti-PD-L1 were also observed even in the presence of the anti-VEGF combination, but no difference was observed compared with anti-PD-L1 alone (Fig. 4b).

Though the percentages of natural killer (NK) cells and Treg cells expressing Ki-67 did not change (Fig. 4c, d, g, h), an increase in GzmB^+^ NK cells and Ki-67^+^ Thconv cells was observed with anti-PD-L1 treatment compared to control (Fig. 4c–f). In addition, GzmB^+^ NK cells were also increased by the combination of anti-PD-L1 and anti-VEGF compared to anti-VEGF alone (Fig. 4c, d). PD-1^+^ Thconv cells were increased by the combination with anti-PD-L1 and anti-VEGF compared to control and anti-VEGF alone (Fig. 4e, f). Although PD-1^+^ Treg cells were statistically significantly increased by the combination with anti-PD-L1 and anti-VEGF compared to control and anti-VEGF alone, the difference was very slight (Fig. 4g, h).

The activation of CD8^+^ T cells, NK cells, and Thconv cells were increased by anti-PD-L1 administration but was not enhanced by the combination of anti-PD-L1 and anti-VEGF.

Among CD45- cells of the whole brain suspension, which includes tumor cells, a subpopulation characterized by high FSC-A indicative of larger cell size, was identified. These cells, which were rarely present in the brain of normal mice, are thought to comprise mostly tumor cells. The expression of PD-L1 on the surface of these cells was detected by FCM analysis (Supplementary Fig. S2).

### ***Increase in the number of activated CD8***^+^***T cells treated with anti-PD-L1 antibody combined with anti-VEGF antibody***

**Fig. 5:**
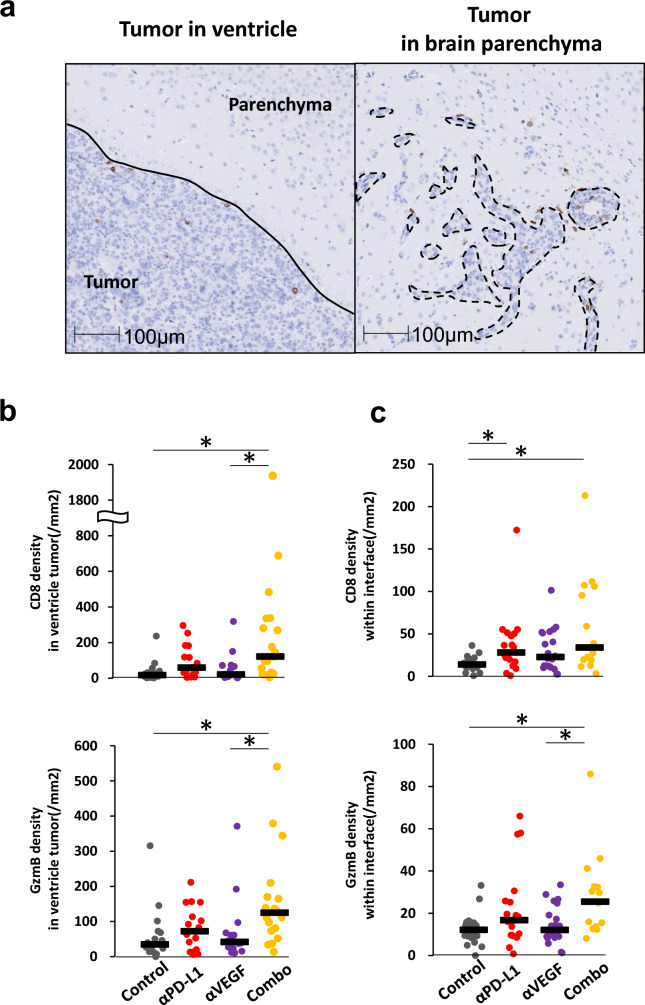
CD8^+^ cells and Granzyme B ^+^ cells localized intertumoral and contiguous peritumoral stroma of the metastatic brain tumor and their densities were increased in the combination group. Anti-PD-L1 antibody (αPD-L1) or mouse IgG (Control) was administered intraperitoneally into a brain metastasis model mice twice a week at a dose of 10 mg/kg. The anti-VEGF antibody (αVEGF) or mouse IgG (Control) was administered intraperitoneally into brain metastasis model mice weekly at a dose of 10 mg/kg. Brains were removed from the mice on day 8, with the drug efficacy study starting on day 1. Three independent experiments were pooled and analyzed. Brain slices were stained by immunohistochemistry using anti-CD8 antibody and anti-Granzyme B antibody. Quantitative analysis of CD8^+^ and GzmB^+^ cell density within the tumor in brain ventricular metastases and within the tumor and the peritumoral area within 500 μm in brain parenchymal metastases was performed. **a** Representative micrographs of CD8^+^ cell in ventricle tumor and brain parenchymal metastases. Scale bar is 100 μm. Black dotted line: Tumor metastases in parenchyma. **b** Quantitative analysis of CD8^+^ and Granzyme B^+^ cell densities in ventricle tumor (n = 18, 17, 16, 19/group). **c** Quantitative analysis of CD8^+^ and Granzyme B^+^ cell densities within the tumor and the peritumoral area within 500 μm in brain parenchymal metastases (n = 20, 19, 20, 15/group). Dots indicate individuals and bars represent median. Statistical differences are shown as *, p < 0.05 (assessed by Wilcoxon’s rank sum test with Holm-Bonferroni correction)

To determine the density and localization of immune cells within each metastatic lesion, we conducted pathological analyses. First, we analyzed effector cell density in ventricular metastases. The ventricular metastatic foci had distinct borders. CD8^+^ cells and GzmB^+^ cells, one of the effector molecules of CD8^+^ T cells and NK cells for cytotoxicity, were mainly located inside foci and few immune cells infiltrated from outside foci (Fig. 5a). Compared to control, the combined anti-PD-L1 and anti-VEGF group showed a statistically significant increase in the density of both CD8^+^ cells and GzmB^+^ cells. There was also trend toward an increase in the density of CD8^+^ cells and GzmB^+^ cells in the anti-PD-L1 compared to control, and in the combined anti-PD-L1 and anti-VEGF group compared to anti-PD-L1 alone (The median density of CD8^+^ cells in the control, anti-PD-L1, and combination groups was 17.296, 59.508 and 121.375, respectively, and the median density of GzmB^+^ cells in the control, anti-PD-L1, and combination groups was 34.565, 72.015, and 124.540, respectively), but no significant difference was observed (Fig. 5b).

Next, we analyzed effector cell density in brain parenchymal metastases. Since CD8^+^ and GzmB^+^ cells infiltrated from parenchyma surrounding the metastases (Fig. 5a), we evaluated the cell density within the tumor and the peritumoral area within 500 μm. An increase in the density of CD8^+^ cells was observed with the administration of anti-PD-L1 compared to control. There was trend toward increase in the density of GzmB^+^ cells in the anti-PD-L1 compared to control but this was not statistically significant (The median density of GzmB^+^ cells in the control, and anti-PD-L1 groups was 12.111 and 16.705). The combined anti-PD-L1 and anti-VEGF group showed significant increase in the density of both CD8^+^ cells and GzmB^+^ cells compared to control. Although trend toward increase was observed in the density of CD8^+^ cells and GzmB^+^ cells in the combined anti-PD-L1 and anti-VEGF group compared to anti-PD-L1 alone (The median density of CD8^+^ cells in the anti-PD-L1, and combination groups was 27.933 and 33.767, respectively, and the median density of GzmB^+^ cells in the anti-PD-L1, and combination groups was16.705, and 25.432, respectively), the difference was not statistically significant (Fig. 5c).

Focusing on the number of activated cells, an increasing trend was observed in the anti-PD-L1, but significant increase was detected only in the anti-VEGF combination group.

### Effect of MVD in the metastatic brain lesions of mice treated with anti-PD-L1 antibody in combination with anti-VEGF antibody

**Fig. 6:**
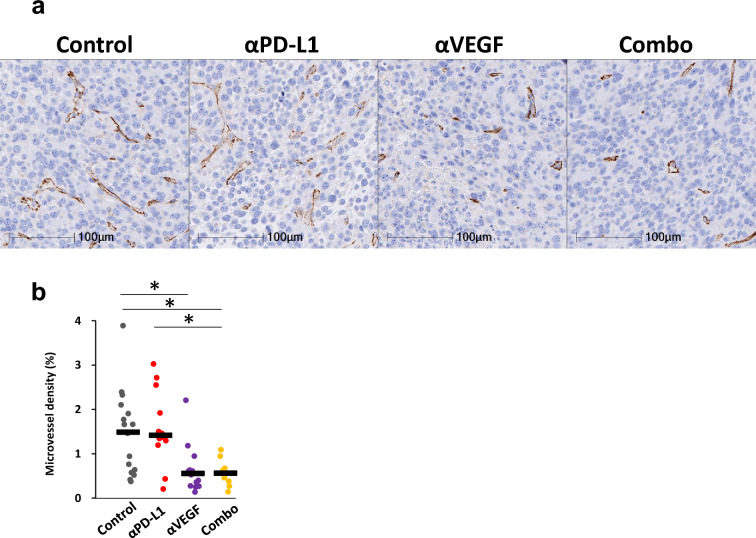
Effects of MVD in ventricle tumor treated with anti-VEGF antibody. Anti-PD-L1 antibody (αPD-L1) or mouse IgG (Control) was administered intraperitoneally into a brain metastasis model mice twice a week at a dose of 10 mg/kg. The anti-VEGF antibody (αVEGF) or mouse IgG (Control) was administered intraperitoneally into brain metastasis model mice weekly at a dose of 10 mg/kg. Brains were removed from the mice on day 8, with the drug efficacy study starting on day 1 (n = 17, 12, 14, 11/group). Three independent experiments were pooled and analyzed. Brain slices were stained by immunohistochemistry using anti-CD31 antibody. Analysis of microvessel density (MVD) in tumor tissue was determined by calculating the ratio of the CD31-positive area to the ventricular metastatic lesions. **a** Representative micrographs of tumor microvessels. Scale bar is 100 μm at higher magnification. **b** MVD in tumor tissues. Dots indicate individuals and bars represent median. Statistical differences are shown as *, p < 0.05 (assessed by Wilcoxon’s rank sum test with Holm-Bonferroni correction)

Since we and others have reported that anti-VEGF antibody leads to the regression of tumor vasculature in subcutaneously established syngeneic tumors [38], we next analyzed the effect of anti-VEGF administration on blood vessels in the current immunocompetent brain metastasis model. Evaluation of intertumoral microvascular density showed that MVD was lower with anti-VEGF alone and in combination with anti-PD-L1 compared to control, and that the effect of anti-PD-L1 was not observed in ventricular metastases (Fig. 6a, b).

As for the brain parenchyma, we could not analyze MVD because there were no mature metastatic foci with neovascularization (data not shown).

In ventricle where mature metastatic foci with neovascularization was observed, and the reduction in MVD in ventricular metastases was confirmed in the anti-VEGF group, suggesting that anti-VEGF suppressed angiogenesis.

## Discussion

In the current study, we established a hematogenous brain metastasis model by inoculating the murine cancer cell line MBT2 into isogenic immunocompetent mice via the internal carotid artery. Although the brain metastasis model established here is based on a single cell line, and further research is needed to enhance the generalizability of the findings, this model allows for the precise evaluation of the anti-tumor activities of ICIs, alone or in combination. Furthermore, we successfully elucidated their modes of action. In the evaluation of total tumor burden in the brain, which was assessed based on whole brain Nluc activity, the combination of anti-PD-L1 and anti-VEGF showed stronger anti-tumor activity than each antibody alone (Fig. 2b). In this model, metastases were observed not only in the brain parenchyma but also in the ventricles. Upon conducting a histopathological examination of the brain metastatic lesions, we found that the ventricular metastases were histologically larger than those in the brain parenchyma (Fig. 1). The luciferase activity measured in the whole brain represents the sum of all metastatic foci; therefore, tumor lesions of the ventricles were thought to contribute more than those of the parenchyma to the total tumor burden and to its differences from the other treatment groups. Since brain metastases are usually parenchymal and metastases in the brain ventricles are rare in clinical settings [39, 40], we attempted to evaluate the antitumor effect of the drug on metastatic lesions in the brain parenchyma and ventricle separately, but it was technically impossible to isolate each tumor lesion from the whole brain. However, as discussed in detail below, since the combined use of anti-PD-L1 and anti-VEGF resulted in an increase in the number of tumor-infiltrating CD8^+^ cells and GzmB^+^ cells—which are generally responsible for cytotoxicity against tumor cells—compared to control, IHC analysis suggested that the potential for anti-tumor activity was increased in both ventricular and parenchymal tumor lesions.

The effect of each antibody and the combination on immune cells was analyzed using two methods, FCM and IHC, with separate aims. Firstly, similar to tumor burden, FCM analysis was conducted for whole brain cell suspension, which includes not only tumor cells, but other cells derived from brain tissues with a variable recovery rate due to aggregation. Therefore, we focused on the proportion of activated cells among CD8^+^ T cells and NK cells. We were able to confirm the enhancement of CD8^+^ T cell and NK cell activation by anti-PD-L1 alone and/or in combination with anti-VEGF in brain (Fig. 4b, d). Secondly, through the IHC staining of pathological sections, we were able to analyze the density of both CD8^+^ cells and GzmB^+^ cells using this method. The ventricles and parenchyma of the tumor were analyzed separately. In the ventricles, an increase in the density of both CD8^+^ cells and GzmB^+^ cells were observed in the group treated with a combination of anti-PD-L1 and anti-VEGF compared to control (Fig. 5b). In the brain parenchyma, an increase in the density of both CD8^+^ cells and GzmB^+^ cells was also observed in the group treated with a combination of anti-PD-L1 and anti-VEGF compared to control (Fig. 5c). In studies using subcutaneously transplanted tumors, it has been shown that the combined blockade of PD-L1/PD-1 axis and VEGF induces an increase in the infiltration of activated CD8^+^ T cells into the tumor either through an increase in the expression of T cell migration factor CXCR3 ligands within the tumor, or through an increase or decrease in the expression of adhesion molecules or FasL, respectively, on vascular endothelial cells in tumor, thereby enhancing anti-tumor effects [38] [41] [42]. In this model, PD-L1 monotherapy did not reach a level where a significant difference could be detected, but the combined use of anti-PD-L1 and anti-VEGF consistently showed a significant increase in the number of CD8^+^ cells and GzmB^+^ cells in the ventricles and brain parenchyma metastases (Fig. 5b, c). This suggests the possibility that the anti-VEGF treatment promotes the infiltration of CD8^+^ T cells activated by anti-PD-L1 into the tumor by enhancing the expression of vascular endothelial adhesion molecules and through the production of chemokines, also in the metastatic brain tumor. Further research will be required to understand the detailed mechanism for why the number of CD8^+^ T cells increased in the brain metastasis model.

In addition, not only CD8^+^ T cell and NK cell activation, but also the enhancement of Thconv cell activation was observed with anti-PD-L1 alone or in combination with anti-VEGF (Fig. 4f).

Regarding Treg cells, most Treg cells were in activated status even in the control group. The ratio of Ki-67^+^ among Treg cells was also high and there was no change observed with antibody treatments (Fig. 4h). Since it ultimately leads to an increase in CD8^+^ T cell activation, our results suggested that CD8^+^ T cell activation overcame the suppressive effect of Treg cells, if any, in the current model. Although previous reports show that anti-VEGF inhibits the proliferation of Treg and reduces intratumoral Treg cells [13], in the current model, the anti-VEGF antibody did not change the ratio of Ki67^+^ Treg cells (Fig. 4h). The difference may be due to the characteristics of the particular model used, such as the tumor cell line or the site of the tumor, but further studies will be needed to clarify the effect of VEGF blockade on Treg proliferation and its suppressive effects. On the other hand, we speculated that it might further enhance the antitumor effect in combination with anti-PD-L1 alone or the anti-PD-L1/anti-VEGF combo if we could achieve the depletion and/or suppression of Treg cells using other agents in this hematogenous brain metastasis model.

In the current study, the activation of CD8^+^ T cells and Thconv cells, as well as an increase in the percentage of activated DCs in live cells, were observed in the CLNs, reported to be draining lymph nodes of brain metastatic tumors, following anti-PD-L1 administration alone (Fig. 3b, d, g). In studies using subcutaneously transplanted tumors, even in tumor models with low PD-L1 expression, it has been shown that blocking PD-L1 expressed on DCs in the lymph nodes promotes the priming of CD8^+^ T cells and induces significant anti-tumor activity [43]. In the current model, PD-L1 is expressed on DCs in lymph nodes (Supplementary Fig. S1), and it is thought that blocking PD-L1 expressed on DCs in the lymph nodes with antibodies could promote priming and enhance anti-tumor activity. In our previous study, in which activated immune cells were transferred to immunodeficient mice with brain metastases, the enhanced anti-tumor effect of anti-PD-L1 was suggested to be due to the intratumoral activation of tumor-infiltrating CD8^+^ T cells [24]. Here, as we expected, using this immunocompetent model, we showed that anti-PD-L1 can activate CD8^+^ T cells both in the metastatic tumor lesion in brain and its tumor-draining lymph nodes, suggesting that the cancer immunity cycle, including the affiliated lymph nodes, is functioning also in metastatic brain tumors. Hung et al. reported that the therapeutic effect of the combination of anti-PD-1 and anti-TIGIT antibody was correlated with both increased effector T cell function and downregulation of suppressive Tregs and tumor-infiltrating DCs using the intracranial injection of murine glioblastoma GL261 [44]. Although Taggart et al., using a mouse model of brain metastasis established by intracranial injection of melanoma cells, reported that the presence of subcutaneously transplanted extracranial tumors and the activation/release of CD8^+^ T cells were required for the anti-PD-1 and anti-CTLA-4 combination to exert its therapeutic effect on intracranial tumors [45], in our model we did not visually detect any extracranial lesions after the intra-carotid artery injection of MBT2 cells. Although there are differences in the models, extracranial tumors are probably unnecessary in this hematogenous brain metastasis model, and the cancer immunity cycle is functioning, including through brain metastases and priming in the regional lymph nodes of the metastases, leading to antitumor effect.

Regarding tumor vascular density, a decrease in tumor microvascular density was detected in the ventricles with the anti-VEGF antibody (Fig. 6b), and we speculated that the anti-tumor effect is enhanced through both the anti-PD-L1 combination-induced increase in the number of immune cells and the anti-angiogenic effect. On the other hand, although no lesions that could be analyzed for MVD were recognized in the brain parenchyma in this model (data not shown), we have confirmed anti-VEGF antibody’s intratumoral penetration in hematogenous models, which worked well in reducing MVD [23].

Based on the results of the phase III IMpower150 study comparing atezolizumab plus bevacizumab plus carboplatin and paclitaxel (ABCP) or atezolizumab plus carboplatin and paclitaxel (ACP) to bevacizumab plus carboplatin and paclitaxel (BCP), ABCP has become a standard-of-care regimen and is approved for the first-line treatment of metastatic non-squamous NSCLC without EGFR/ ALK genetic alterations (United States and Europe). In the final exploratory analysis of the IMpower150 study, it was reported that the development of new brain metastases was delayed further by ABCP compared to BCP [46]. Furthermore, in the real-world data analysis of IMpower150, intracranial response rates were over 50% regardless of whether symptomatic or asymptomatic [47]. However, the mechanism of anti-PD-L1 and anti-VEGF against metastatic brain tumors is still not clarified. Further analysis of the anti-tumor effects of ICIs and VEGF inhibitor combinations on brain metastases is needed.

## Supplementary Information

Below is the link to the electronic supplementary material.

Supplementary file1 (PPTX 616 KB)

## Data Availability

The datasets generated during and/or analyzed during the current study are available from the corresponding author on reasonable request.

## Abbreviations

BBB: Blood–Brain Barrier
BTB: Blood–Tumor Barrier
CLNs: Cervical Lymph Nodes
DC: Dendritic Cell
FCM: Flow Cytometry
ICIs: Immune Checkpoint Inhibitors
IgG: Immunoglobulin G
IHC: Immunohistochemistry
mAb: Monoclonal Antibody
MDSC: Myeloid Derived Suppressor Cell
MVD: Microvessel Density
NK: Natural Killer
NSCLC: Non-Small Cell Lung Cancer
PD-1: Programmed Death-1
PD-L1: Programmed Death-Ligand 1
RLU: Relative Light Unit
Thconv: Conventional Helper T
Treg: Regulatory T
VEGF: Vascular Endothelial Growth Factor
