# Supplementary material for: PD-L1 and VEGF dual blockade enhances anti-tumor effect on brain metastasis in hematogenous metastasis model
Source: Clin Exp Metastasis. 2024 Sep 5;41(6):909–24. doi: 10.1007/s10585-024-10309-y (PMC11607052; doi:10.1007/s10585-024-10309-y)
Supplement: Supplementary file 1 — Supplementary file1 (PPTX 616 KB) [file 10585_2024_10309_MOESM1_ESM.pptx]

## Slide 1
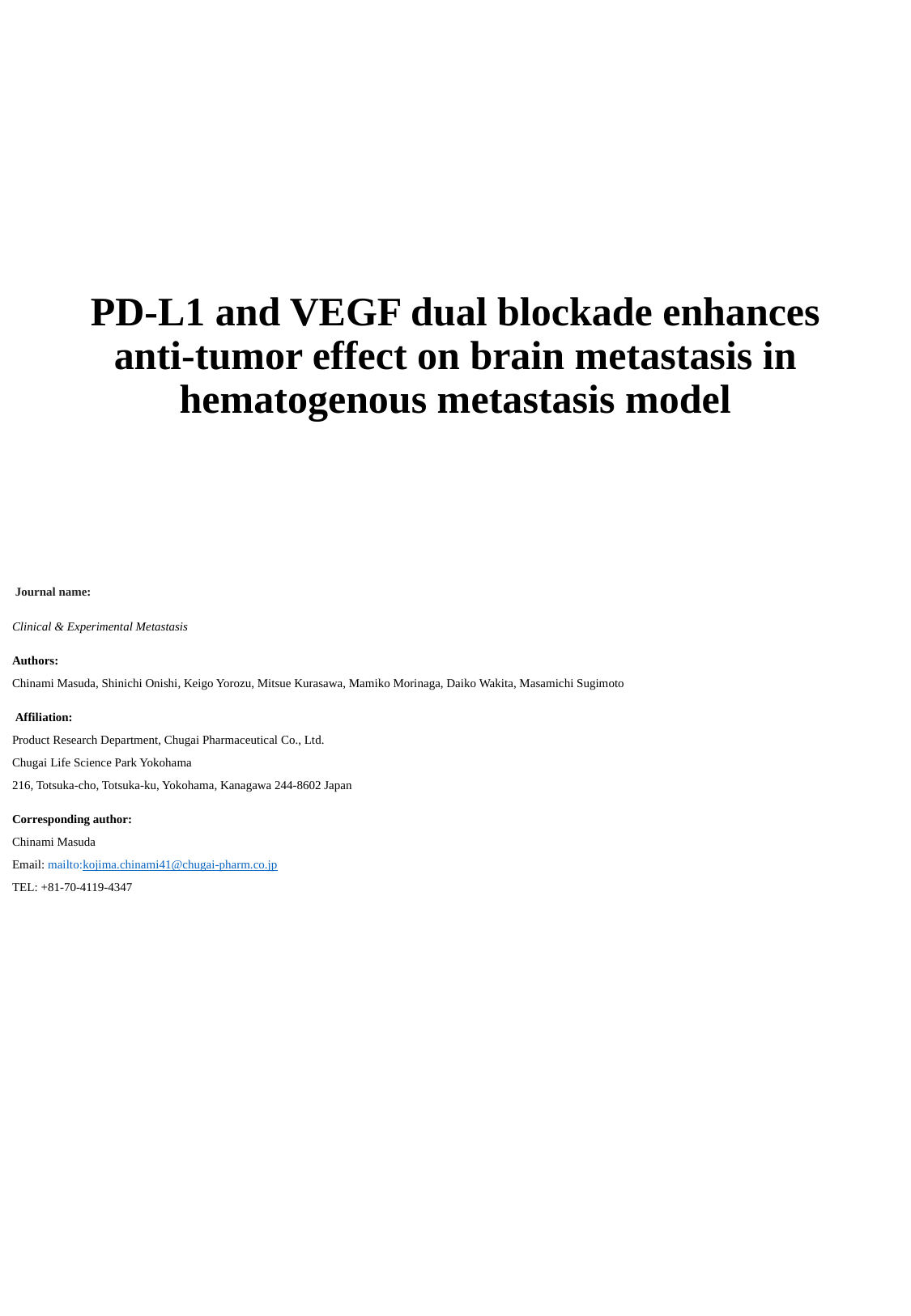

# PD-L1 and VEGF dual blockade enhances anti-tumor effect on brain metastasis in hematogenous metastasis model
 Journal name:
Clinical & Experimental Metastasis
Authors:
Chinami Masuda, Shinichi Onishi, Keigo Yorozu, Mitsue Kurasawa, Mamiko Morinaga, Daiko Wakita, Masamichi Sugimoto
 Affiliation:
Product Research Department, Chugai Pharmaceutical Co., Ltd.
Chugai Life Science Park Yokohama
216, Totsuka-cho, Totsuka-ku, Yokohama, Kanagawa 244-8602 Japan
Corresponding author:
Chinami Masuda
Email: mailto:kojima.chinami41@chugai-pharm.co.jp
TEL: +81-70-4119-4347

## Slide 2
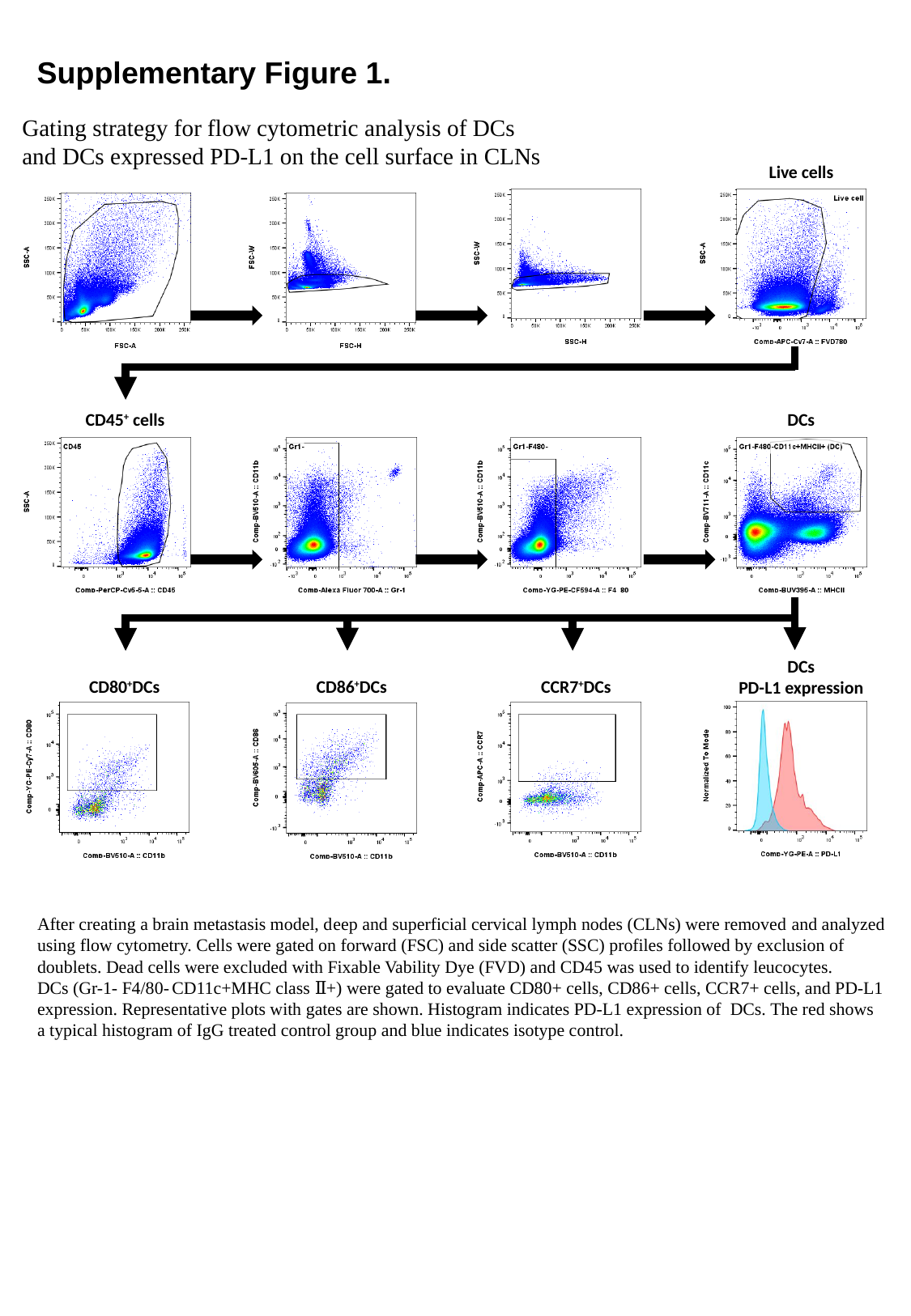

Supplementary Figure 1.
Gating strategy for flow cytometric analysis of DCs
and DCs expressed PD-L1 on the cell surface in CLNs
Live cells
CD45+ cells
DCs
DCs
PD-L1 expression
CD80+DCs
CD86+DCs
CCR7+DCs
After creating a brain metastasis model, deep and superficial cervical lymph nodes (CLNs) were removed and analyzed using flow cytometry. Cells were gated on forward (FSC) and side scatter (SSC) profiles followed by exclusion of doublets. Dead cells were excluded with Fixable Vability Dye (FVD) and CD45 was used to identify leucocytes.
DCs (Gr-1- F4/80- CD11c+MHC class Ⅱ+) were gated to evaluate CD80+ cells, CD86+ cells, CCR7+ cells, and PD-L1 expression. Representative plots with gates are shown. Histogram indicates PD-L1 expression of  DCs. The red shows a typical histogram of IgG treated control group and blue indicates isotype control.

## Slide 3
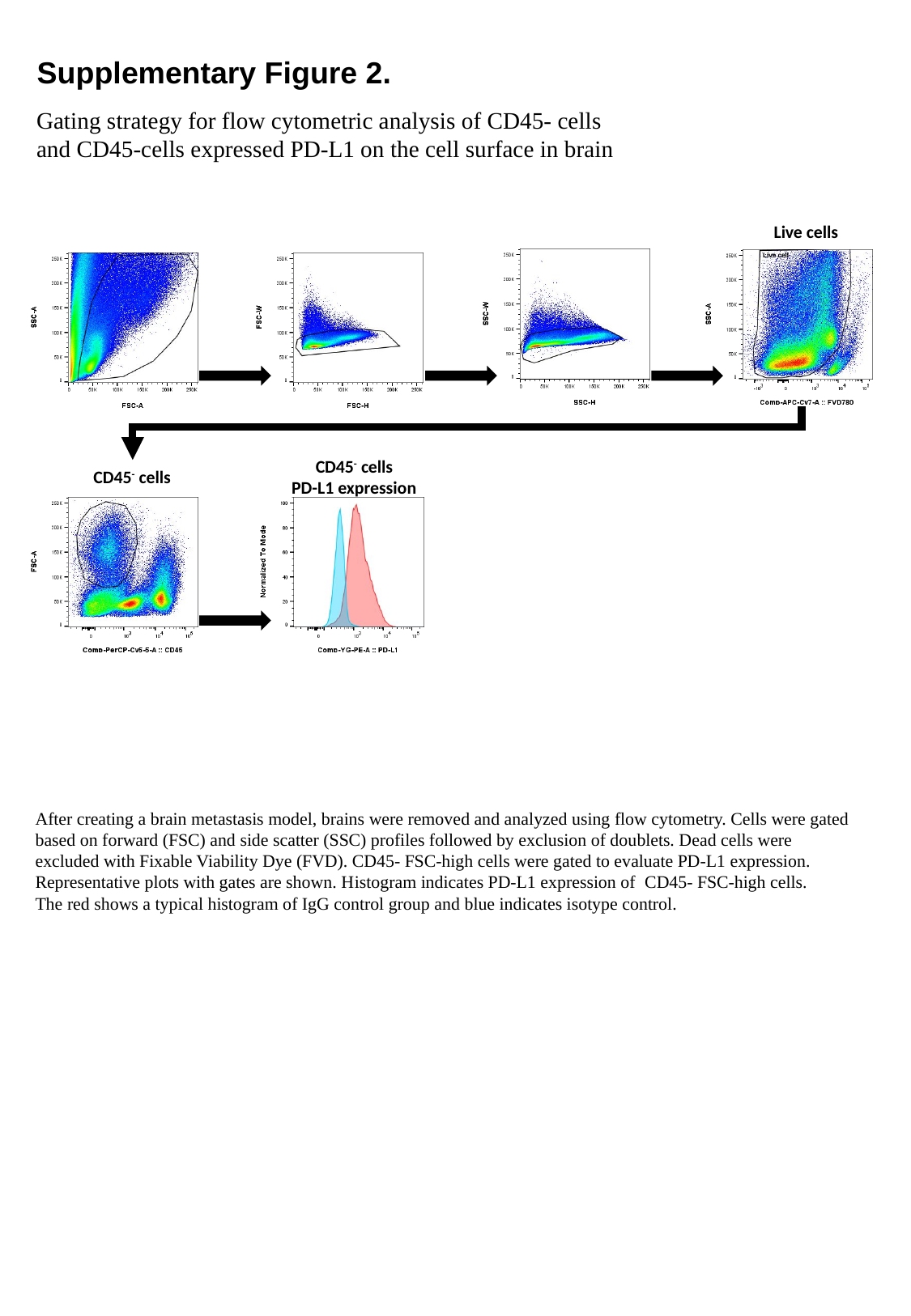

Supplementary Figure 2.
Gating strategy for flow cytometric analysis of CD45- cells
and CD45-cells expressed PD-L1 on the cell surface in brain
Live cells
Live cell
CD45- cells
PD-L1 expression
CD45- cells
After creating a brain metastasis model, brains were removed and analyzed using flow cytometry. Cells were gated based on forward (FSC) and side scatter (SSC) profiles followed by exclusion of doublets. Dead cells were excluded with Fixable Viability Dye (FVD). CD45- FSC-high cells were gated to evaluate PD-L1 expression. Representative plots with gates are shown. Histogram indicates PD-L1 expression of  CD45- FSC-high cells.
The red shows a typical histogram of IgG control group and blue indicates isotype control.

## Slide 4
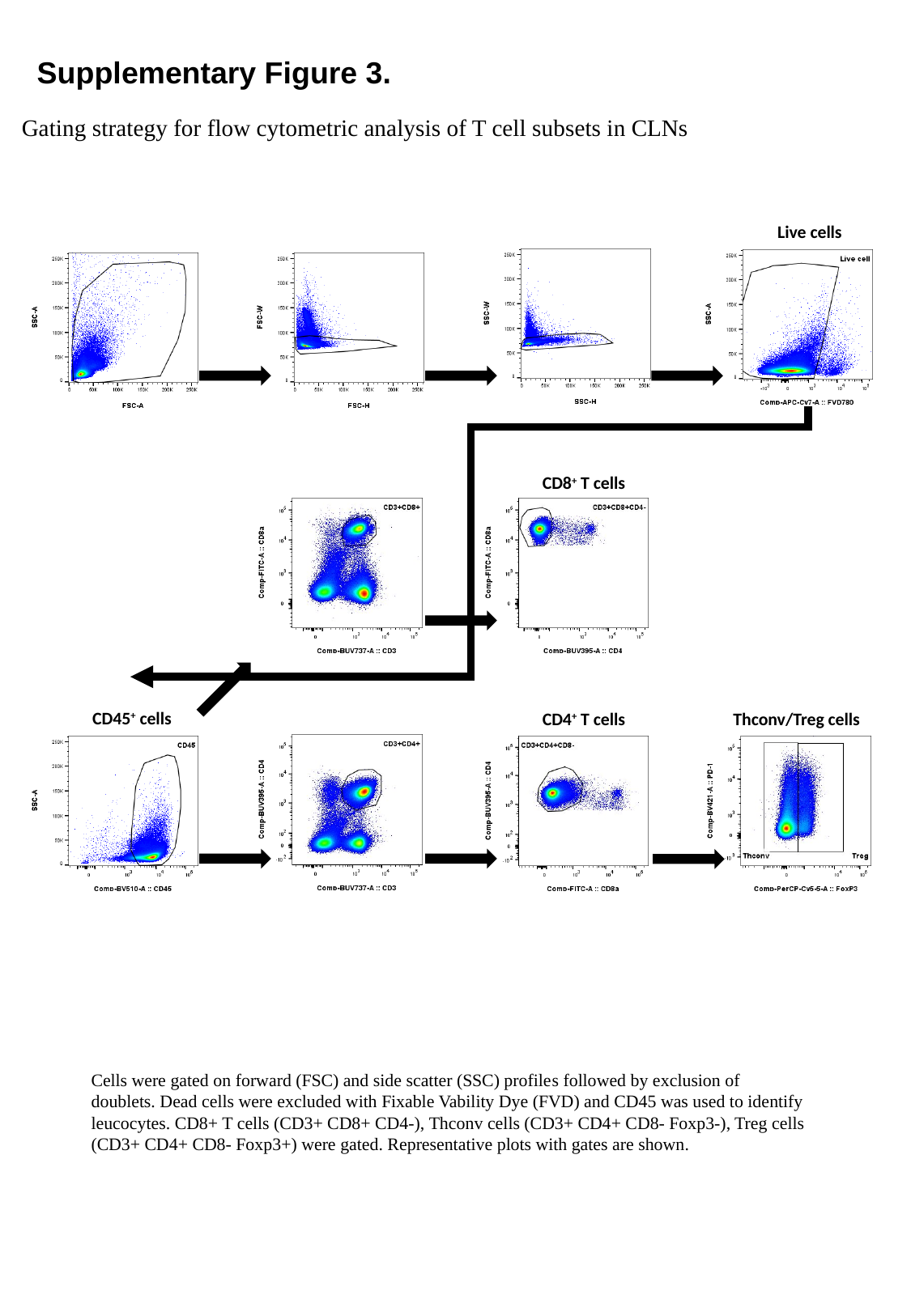

Supplementary Figure 3.
Gating strategy for flow cytometric analysis of T cell subsets in CLNs
Live cells
CD8+ T cells
CD45+ cells
CD4+ T cells
Thconv/Treg cells
Cells were gated on forward (FSC) and side scatter (SSC) profiles followed by exclusion of doublets. Dead cells were excluded with Fixable Vability Dye (FVD) and CD45 was used to identify leucocytes.​ CD8+ T cells (CD3+ CD8+ CD4-), Thconv cells (CD3+ CD4+ CD8- Foxp3-), Treg cells (CD3+ CD4+ CD8- Foxp3+) were gated. Representative plots with gates are shown.

## Slide 5
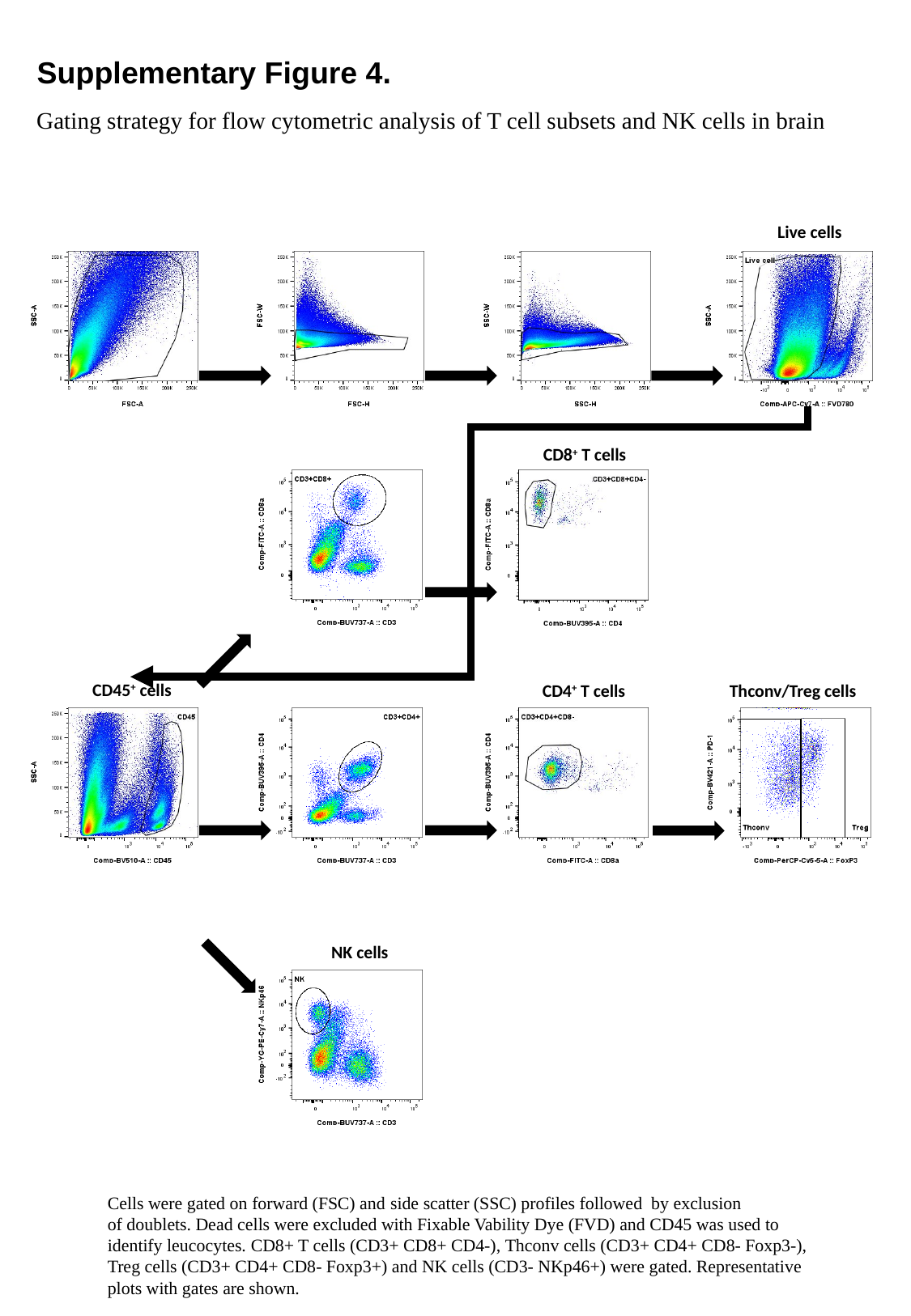

Supplementary Figure 4.
Gating strategy for flow cytometric analysis of T cell subsets and NK cells in brain
Live cells
CD8+ T cells
CD45+ cells
CD4+ T cells
Thconv/Treg cells
NK cells
Cells were gated on forward (FSC) and side scatter (SSC) profiles followed  by exclusion of doublets. Dead cells were excluded with Fixable Vability Dye (FVD) and CD45 was used to identify leucocytes.​​ CD8+ T cells (CD3+ CD8+ CD4-), Thconv cells (CD3+ CD4+ CD8- Foxp3-), Treg cells (CD3+ CD4+ CD8- Foxp3+) and NK cells (CD3- NKp46+) were gated. Representative plots with gates are shown.
